# Mindfulness in Facilitating Pelvic Floor Botulinum Toxin Injection in Women with Chronic Pelvic Pain

**DOI:** 10.3390/toxins16050216

**Published:** 2024-05-09

**Authors:** Jacqueline V. Aredo, Hannah K. Tandon, Samin Panahi, Vy T. Phan, Rezvan Ameli, Barbara I. Karp, Pamela Stratton

**Affiliations:** 1 Rehabilitation Medicine Department, Intramural Research Program, Clinical Center, National Institutes of Health, Bethesda, MD 20892, USA; jaredo@stanford.edu (J.V.A.); vtp9@georgetown.edu (V.T.P.); 2 Department of Medicine, University of California, San Francisco, CA 94143, USA; 3 Department of Internal Medicine and Geriatrics, Oregon Health and Science University, Portland, OR 97239, USA; 4 Department of Family Science, School of Public Health, University of Maryland, College Park, MD 20742, USA; 5 School of Medicine, Georgetown University, Washington, DC 20007, USA; 6 NIMH Representative to the Clinical Center Pain and Palliative Care Service, National Institutes of Health, Bethesda, MD 20892, USA; rezvan.ameli@gmail.com; 7 Office of the Clinical Director, Intramural Research Program, National Institute of Neurological Disorders and Stroke, National Institutes of Health, Bethesda, MD 20892, USA; karpb@ninds.nih.gov

**Keywords:** chronic pelvic pain, botulinum toxin, mindfulness, officed-based procedures, complementary medicine

## Abstract

Botulinum toxin (BoNT) injection can safely be done as an office-based procedure, but can be painful itself, especially when injecting pelvic floor muscles to treat chronic pelvic pain (CPP). Mindfulness interventions may reduce procedure-associated acute anxiety and pain. We applied mindfulness techniques to increase the tolerability of office-based pelvic floor BoNT injections in women with CPP. Women enrolled in a clinical trial of BoNT for endometriosis-associated CPP were offered a brief, guided mindfulness session before and/or after transvaginal injection. Anxiety, pain, and dysphoria were rated on a 0–10 numerical rating scale (NRS) before and after each mindfulness session. Eight women underwent mindfulness sessions. Five participants had a session before and two after the transvaginal injection. One participant had two sessions: one before and one after separate injections. All six women completing a session prior to injection had at least moderate anxiety, which lessened after the mindfulness session (median NRS change: −3.3/10). All three women reporting injection-associated pain experienced less intense pain following the post-injection session (median NRS change: −3/10). Three women experiencing dysphoria improved after the session (median NRS change: −3/10). A brief, guided mindfulness session may lessen acute pain, anxiety, and dysphoria associated with office-based transvaginal BoNT injection.

## 1. Introduction

When used to treat chronic pelvic pain (CPP) in women, botulinum toxin (BoNT) is most frequently injected into pelvic floor muscles such as the levator ani and obturator internus [1]. Acute pain and anxiety often accompany such gynecologic procedures due to the inherently intrusive nature of the pelvic examination and the injections themselves, most often done through a transvaginal or transperineal approach. Pain sensitization, previously demonstrated in women with chronic pelvic pain, as well as anxiety associated with the anticipation of increased pain from the procedure, further exacerbates the discomfort and pain experienced [2]. While performing BoNT injection procedures under anesthesia would minimize procedural pain, general anesthesia eliminates the injector’s ability to identify spasms in the affected muscles during the procedure and imposes additional medical risk as well as increased cost, staff, and inconvenience. Thus, techniques that could mitigate anxiety and pain would enhance patient comfort and facilitate the conduct of these painful procedures in the outpatient setting.

Mindfulness is defined as “the awareness that arises by paying attention on purpose, in the present moment, and nonjudgmentally to the unfolding of experience” [3]. Mindfulness has arisen primarily from the Buddhist practices of meditation which spread throughout the United States in the mid-twentieth century. In 1979, Dr. Jon Kabat-Zinn developed his mindfulness-based stress reduction program, which was initially used for stress management, but has now shown broader impacts in improving various health-related disorders [4]. Mindfulness interventions are effective complementary approaches to the management of chronic pain [5,6]. From a mechanistic standpoint, mindfulness practices have been shown to ameliorate the pain response through top-down inhibition of ascending nociceptive signals, with multiple processes involving the activation of the primary somatosensory cortex as well as thalamic deactivation through GABAergic neurons [7,8]. Mindfulness-based stress reduction programs for chronic conditions typically consist of about 30 h of patient training and practice and have evidence-based demonstrated benefits for pain, anxiety, and depression [3,9,10,11]. Shorter intervention programs can also be effective [12,13,14]. There are similar data to support that brief peri-procedural mindfulness interventions may be effective in moderating anxiety or stress during office-based medical procedures [15,16,17,18].

Herein, we present our experience using a brief mindfulness session to facilitate transvaginal pelvic floor muscle injection in women enrolled in a clinical trial of BoNT treatment for CPP.

## 2. Results

Eight participants completed nine mindfulness sessions (Table 1). Five participants had a single session before the procedure and two participants had a single session after the procedure; one participant had two sessions, one before and one after procedures that occurred on different days. The median age of these participants was 26 years (range 18–47) and the median duration of pelvic pain was 9.5 years (range 4–20; Table 1). Five of the eight (63%) participants had a history of anxiety and two (25%) had a history of depression. All participants had pelvic floor muscle spasms on examination and seven (88%) had hyperalgesia and allodynia beyond the pelvis, consistent with sensitization.

**Figure 1:**
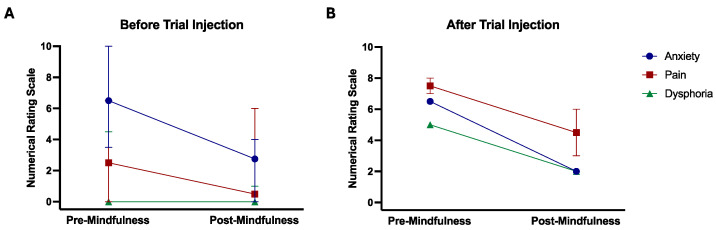
Pre- and post-mindfulness session numerical rating scale scores for anxiety, pain, and dysphoria. Panel (**A**) and panel (**B**) depict the median and range of scores for those who had a mindfulness session before and after transvaginal pelvic floor muscle injection, respectively. The mindfulness session was before transvaginal pelvic floor muscle injection in 5 participants, after injection in 3, and both before and after the procedure in separate injection sessions.

All six participants who completed the mindfulness session before transvaginal injection had marked pre-procedure anxiety (median numerical rating scale [NRS]: 5.8/10; Table 2, Figure 1A). Following the session, NRS decreased (median change 3.3/10), with two participants reporting a resolution of anxiety. Three participants with moderate pre-session pain (median NRS: 6/10) experienced less pain after the mindfulness session (median NRS change: −3/10). Two participants also had baseline dysphoria (median NRS: 3.8/10) lessened after the session (median NRS change: −3.3/10), with dysphoria resolving completely in one participant.

Three participants completed the mindfulness session after transvaginal injection (Table 2; Figure 1B). All had intense pain post-procedure (median NRS: 7.5/10), with one participant unable to rate her pain level. All had less intense pain after the mindfulness session (median NRS change: −3/10). Post-procedure anxiety, initially present in all three participants, improved in two. The one participant with dysphoria had improved after the session. Data on dysphoria were missing for the other two participants.

## 3. Discussion

In this small cohort of women with CPP, a brief, guided mindfulness session before and/or after an office-based transvaginal injection of botulinum toxin reduced self-reported procedural anxiety, pain, and dysphoria. In these patients, procedural pain is often exacerbated by several factors. First, these patients often have a history of dyspareunia and have apprehension about experiencing injection through an extremely sensitive area into their most tender sites, typically areas of spasm, in the pelvic floor muscles. In our patients, the injections are targeted into areas of pelvic floor muscle spasm, where muscle palpation elicits the patients’ typical pelvic pain. In addition, several of these patients had a history of comorbid anxiety and depression for which they were receiving treatment. Such co-morbid psychiatric conditions have been shown to worsen painful experiences in patients who suffer from chronic pain [19,20,21,22]. Furthermore, almost all of our cohort had evidence of central sensitization that reduces pain thresholds and amplifies the perception of noxious stimuli [2,23]. Mechanistically, procedural anxiety and catastrophizing can create a pro-inflammatory stress response that further intensifies pain in the office procedural setting [24]. Yet, in this group of patients with multiple converging factors that may increase pain, we found that a brief mindfulness intervention was able to reduce pre- and/or post-transvaginal injection anxiety and pain beyond that achieved with an oral anxiolytic and intravaginal topical analgesic. These results suggest that mindfulness is of potential benefit and could facilitate tolerance of office-based transvaginal pelvic floor BoNT injection, and perhaps other painful gynecological and nongynecological toxin injection procedures.

Brief peri-procedural mindfulness interventions have been previously evaluated in women undergoing breast biopsy. One randomized clinical trial identified a significant reduction in anxiety during the procedure among women who were guided in a mindfulness meditation session before the biopsy compared to women who solely had guided focused breathing or standard care [15]. However, no differences in pain during the procedure were observed. Another randomized clinical trial reported lower perceived stress following biopsy among women participating in a series of mindfulness sessions compared to controls, but no group differences were observed in depression, anxiety, or pain scales [16]. Notably, this latter study only examined these outcome measures at a single time point and did not compare between-group differences before and after the mindfulness intervention or before and after the biopsy procedure. In contrast, an eight-week mindfulness program was evaluated in a pilot study of women with chronic pelvic pain and showed significant improvement in maximum daily pain scores, physical and social function, and mental health, suggesting a potential therapeutic component to mindfulness in chronic pelvic pain, even in the absence of a painful procedure [25].

Our experience suggests that a single guided mindfulness session can be incorporated as a supplemental intervention alongside standard-of-care conscious sedation or anxiolytic and topical analgesia for office-based pelvic floor BoNT injections. Additional interventions for pain reduction—such as localized heat or cooling and other environmental modifications (e.g., changes in sound or lighting)—could be easily incorporated alongside the mindfulness practice, although the additive effect of these interventions will need to be evaluated through further study. Our participants’ mindfulness sessions were guided by a clinical psychologist; however, to implement this intervention within a medical practice, a professional (such as a nonphysician provider) could be trained as a mindfulness instructor. Telemedicine may also provide a venue for incorporating such expert input and providing mindfulness guidance to patients.

One limitation of this study is that the women undergoing the mindfulness sessions were self-selected. It is possible that those who were amenable to trying mindfulness with injection were more likely to benefit than the broader population of women with chronic pelvic pain or patients undergoing BoNT injection. Mindfulness interventions have been found to reduce perceived pain and decrease pain catastrophizing among patients with chronic pelvic pain from a variety of etiologies [26,27]; however, the application of mindfulness interventions towards facilitating BoNT injection in this broader patient population remains unexplored, and further inquiry is warranted. Another limitation is the small size of this convenience sample in a pilot substudy, which precluded statistical analysis. We reported all available post-intervention changes, summarizing our observations quantitatively when possible. This analysis describes the immediate effects after the mindfulness intervention; long-term impacts on patient outcomes should be evaluated in subsequent studies. This pilot substudy lacked a control group; we did not assess anxiety, pain, and dysphoria pre- and post-injection in those participants in the clinical trial who declined the mindfulness sessions. Thus, the efficacy of the mindfulness intervention cannot be definitively concluded, but the pre-post differences in each symptom domain in this analysis support further investigation. Additional studies could determine if similar benefits could be obtained with mindfulness interventions in the broader population of patients experiencing pain with BoNT injections and could explore different mindfulness intervention paradigms.

## 4. Conclusions

We found that a brief, guided mindfulness intervention session could be a useful, minimal-risk, complementary aid in facilitating transvaginal BoNT injection in women with chronic pelvic pain. This approach may be more broadly applicable to women undergoing gynecological procedures or for those with high anticipatory/procedural anxiety or pain when undergoing BoNT injection for any indication. Further mindfulness studies are warranted.

## 5. Materials and Methods

### 5.1. Clinical Trial Enrollment and Setting

Women aged 18 to 50 years with pelvic pain persisting after surgical and hormonal treatment of endometriosis were enrolled in a clinical trial of BoNT for their CPP (NCT01553201). Details on eligibility criteria have been previously reported [28]. In brief, women were excluded if their CPP could be primarily attributed to a non-gynecologic cause, if they had untreated severe cervical dysplasia, a history of urinary or fecal incontinence, known pelvic prolapse, or if they had undergone hysterectomy and bilateral salpingo-oophorectomy. The clinical study was approved by the Eunice Kennedy Shriver National Institute of Child Health and Human Development Institutional Review Board and informed consent was obtained from all participants. All study procedures were performed at the Clinical Center of the National Institutes of Health in Bethesda, Maryland.

At enrollment, all women reported their medical history and underwent palpation of the bilateral pubococcygeus, iliococcygeus, and obturator internus muscles to identify areas of spasm, especially those areas that would provoke the patient’s typical pelvic pain, as previously described [28]. All women also underwent a neuro-musculoskeletal examination to evaluate allodynia and hyperalgesia as evidence of peripheral and central nervous system sensitization.

**Figure 2:**
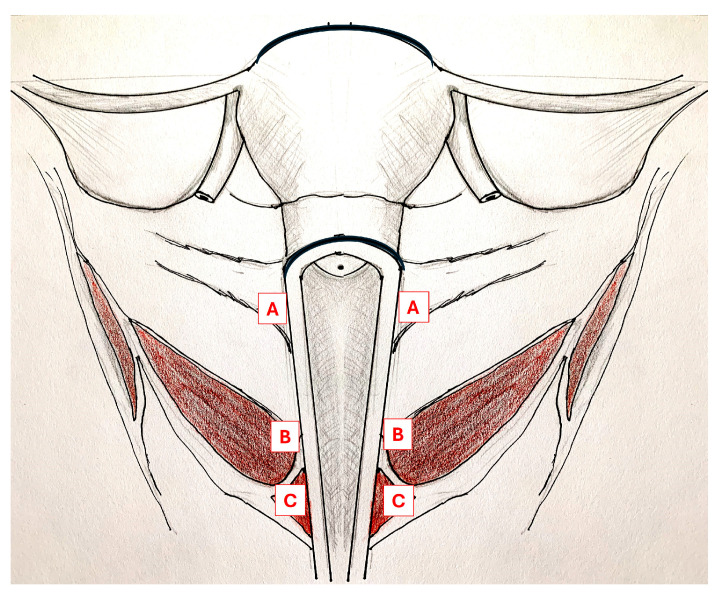
Coronal cross-section of the female pelvis. Anatomic drawing of the female pelvis showing pelvic floor muscles targeted for transvaginal injection which include obturator internus, lateral to the uterine cervix (**A**), iliococcygeus, the lateral vaginal walls (**B**), and pubococcygeus, around the vaginal opening (**C**).

The clinical trial incorporated one randomized injection of BoNT or saline placebo and an additional optional open injection of active drug. OnabotulinumtoxinA (100 units in 4 cc normal saline) or saline (4 cc) was injected into three to four areas of pelvic floor muscle spasm that evoked the participant’s typical pelvic pain on palpation. A single-digit pelvic examination identified areas of tenderness and spasm that elicited each individual’s typical pelvic pain in the bilateral pubococcygeus, iliococcygeus, and obturator internus muscles (Figure 2). The areas of greatest spasm were selected for injection. Injections were administered by a gynecologist under electromyography guidance, as previously described [28]. Participants received 5–10 mg oral diazepam and 4% lidocaine cream was applied to the vaginal mucosa before transvaginal injection. After observing that an on-the-spot, incidental mindfulness session helped a participant with significant procedural anxiety and pain, we offered subsequent participants a mindfulness session before and/or after the trial injections at their option.

### 5.2. Mindfulness Session

Pre-injection mindfulness sessions were conducted prior to diazepam administration. A clinical psychologist provided individualized training and guidance that incorporated two primary mindfulness techniques: body scan and mindful breathing. Body scan uses the systematic shift of attention to individual parts of the body with the goal of directing awareness to each body region and an open and accepting attitude [29]. For mindful breathing, participants use the flow of inspiratory and expiratory breathing as an anchor to focus attention on the present moment [29]. Both techniques are based on mindfulness principles that require focused attention and an accepting attitude toward felt experiences. Each session included an introduction to mindfulness (10 min) followed by instructions on body scan (10 min) and mindful breathing (10 min) for a total practice duration of 20 min. Participants self-reported their level of anxiety, pain, and dysphoria on a numerical rating scale (NRS) ranging from 0 (none) to 10 (worst) intensity at the beginning and conclusion of the mindfulness practice. NRS scores were recorded by the clinical psychologist.

Results for this small pilot substudy are descriptive only and are reported as median NRS score and median change with intervention. The small sample size precluded statistical analysis.

## Figures and Tables

**Table 1:** Participant characteristics.

Characteristic	Result (N = 8)
Age (years), median (range)	26 (18–47)
Duration of pelvic pain (years), median (range)	9.5 (4–20)
History of anxiety, N (%)	5 (63)
History of depression, N (%)	2 (25)
Pelvic floor muscle spasm on examination, N (%)	8 (100)
Hyperalgesia and/or allodynia on examination, N (%)	7 (88)

**Table 2:** Pre- and post-mindfulness session numerical rating scale scores for anxiety, pain, and dysphoria. The mindfulness session was before transvaginal pelvic floor muscle injection in 5 participants, after injection in 3, and both before and after injection in 1.

Subject ID	Pre-Mindfulness			Post-Mindfulness			Difference		
	Anxiety	Pain	Dysphoria	Anxiety	Pain	Dysphoria	Anxiety	Pain	Dysphoria
Before Trial Injection									
1	3.5	6.5	4.5	0	6	0	−3.5	−0.5	−4.5
2	5.5	0	0	2.5	0	0	−3	0	0
3	6	0	0	4	0	0	−2	0	0
4	7	5	3	4	1	1	−3	−4	−2
5	9	0	NA	3	0	NA	−6	0	NA
6 ^a^	10	6	0	0	3	0	−10	−3	0
Median	5.8	2.5	0	2.8	0.5	0	−3.3	−0.3	0
After Trial Injection									
6 ^a^	6.5	8	5	2	6	2	−4.5	−2	−3
7	NA	7	NA	Unchanged	3	NA	Unchanged	−4	NA
8	NA	NA	NA	Decreased	Decreased	NA	Decreased	Decreased	NA
Median	6.5	7.5	5	2	4.5	2	−4.5	−3	−3

Abbreviations: ID identification, NA not available. ^a^ Patient completed mindfulness sessions before and after two different transvaginal injections.

## Data Availability

The data presented in this study are available on request from the corresponding author.
